# *In situ* growth of ZnO nanoparticles in precursor-insensitive water-in-oil microemulsion as soft nanoreactors

**DOI:** 10.1186/s11671-015-0730-9

**Published:** 2015-01-28

**Authors:** Ali Bumajdad, Metwally Madkour

**Affiliations:** Chemistry Department, Faculty of Science, Kuwait University, P.O. Box 5969, Safat, 13060 Kuwait

**Keywords:** Cationic surfactant, Microemulsion, Nanoreactor, Nanostructures, Zinc oxide

## Abstract

Zinc oxide (ZnO) nanostructures of uniform shapes and sizes (spherical, needle-like, and acicular) were directly synthesized using a relatively precursor-insensitive water-in-*n*-heptane microemulsion system stabilized by a mixture of cationic and non-ionic surfactants. With this colloidal system, the synthesized ZnO possesses the highest reported surface area (76 m^2^ g^−1^) among the published reports utilizing other microemulsion systems. Such precursor insensitivity allowed studying the effect of Zn precursor:precipitating agent molar ratio (as high as 1:8) on the particle size, specific surface area, porosity, and morphology of the synthesized nanoparticles. The interaction of the cationic surfactant head groups and their Br^−^ counter ions with Zn^2+^ and OH^−^ ions is believed to play a major role in controlling the ZnO characteristics. Due to such interactions, it is believed that the nucleation processes are retarded while the growth is more dominating if compared with other microemulsion systems.

## Background

Although it has been demonstrated that water-in-oil microemulsion (W/O μ) method is an efficient medium for the preparation of monodispersed functional oxide nanoparticles (NPs), only few studies employed such medium for the synthesis of ZnO NPs, all of which were using anionic or nonionic surfactants [1-4]. Due to the microemulsion destabilization, the maximum Zn precursor:precipitating agent molar ratio studied before was 1:2 or 1:4 for NaOH and NH_4_OH, respectively [1-4]. For example, it was found that the microemulsion destabilized upon using NaOH at 0.525 M [1], whereas in this study, a value of 0.8 M was tested with no obvious destabilization.

In this communication, the preparation of ZnO NPs of different shapes inside the dispersed nano-water droplets was investigated using the W/O μ system of composition (DDAB + Brij®35/*n*-heptane/water). Such microemulsion system was not employed before for the synthesis of ZnO NPs. Using small-angle neutron scattering technique and phase behavior studies, such surfactant mixture was found to produce a minimum droplet interaction and result in a much lower sensitivity toward precursor addition [5,6]. The minimum interaction insured spherical shape of the droplets and low microemulsion viscosity, which would enhance the dynamic nature of the microemulsion droplets. The low precursor insensitivity, however, enabled us to study the effect of varying the Zn precursor:precipitating agent molar ratio without destabilization of the microemulsion. Such effect was studied in details in an aqueous system [7], and this work aims to fill the literature gap for W/O μ system.

## Methods

In a typical synthesis, two microemulsion (μ1 and μ2) systems were prepared from a mixture of water, *n*-heptane, and surfactants (90:10% molar ratio of the double-tailed cationic DDAB (didodecyldimethylammonium bromide):the single-tailed non-ionic Brij®35 (Sigma-Aldrich, St. Louis, MO, USA) at water-to-surfactant molar ratio of *W* = 18. The total surfactant concentration was 0.2 M. For μ1, the aqueous phase contains the precipitating agent, NaOH, with concentrations 0.1, 0.2, 0.4, and 0.8 M. For μ2, the aqueous phase contains the precursor, Zn(NO_3_)_2_, with a fixed concentration of 0.1 M. In a typical procedure, equal volumes of the two microemulsion systems μ1 and μ2 were mixed, giving a transparent microemulsion. Afterwards, the mixture was refluxed 16 h at *T* = 60°C which results in a turbid solution. The mixture then is centrifuged, and the precipitate (designated hereafter as ***Z***1, ***Z***2, ***Z***4, and ***Z***8 for 0.1, 0.2, 0.4, and 0.8 M NaOH, respectively) was washed several times with a mixture of acetone and water and then dried at 110°C overnight.

Thermogravimetric analysis (TGA) was performed on 10- to 15-mg portion of test materials using a Shimadzu TGA-50 thermogravimetric analyzer (Shimadzu Scientific Instruments, Kyoto, Japan) under nitrogen atmosphere in the temperature range 20°C to 800°C with a heating rate of 10°C min^−1^. X-ray diffraction (XRD) measurements were conducted by using Siemens D-5000 (Siemens AG, Munich, Germany) with copper target and nickel filter with CuKα radiation (*λ* = 0.154056 nm). The morphology of the ZnO NPs was obtained by transmission electron microscopy (TEM) using a JEOL JEM 1230 (JEOL Ltd., Tokyo, Japan) operating at 120 KV. The powders were dispersed by ultrasonication in suitable solvent for 3 min before deposition on the TEM grid. Brunauer-Emmett-Teller (BET) surface area was calculated using a model ASAP 2010 automatic Micromeritics sorptiometer (Micromeritics Instrument Corporation, Norcross, GA, USA) equipped with an outgassing platform. X-ray photoelectron spectroscopy (XPS) was conducted using a model Thermo ESCA Lab 250Xi (Thermo Fisher Scientific Inc., MA, USA) equipped with MgKα radiation (1,253 eV) and operated at 23 kV and 13 mA.

## Results and discussion

The process of nucleation and growth of nanoparticles inside the W/O μ droplets starts with droplet collision, coalescence, and then exchange of their contents [8]. This exchange is too rapid and precipitation reaction occurs inside the nanodroplets, which is followed by nucleation, growth, and coagulation of the primary particles, resulting in the formation of the final nanoparticles. The cationic head group of surfactant is believed to play a role in the formation and aggregation behavior of the nanoparticles. The cationic head group attracts the hydroxyl ions and forces the Zn^+2^ to stay at the droplet center. Also, the relatively large Br^−^ ions are expected to have higher binding tendency to Zn^2+^ over that of the small OH^−^ ions (see, for example, [9] for micellar systems and [10] for microemulsion system). Since the ZnBr_2_ solubility is very high (447 g per 100 ml of H_2_O at 20°C [11]) and the Br^−^ ion concentration in the studied system was calculated to be 2.9 g per 100 g of H_2_O which is much less than the solubility limit, hence, ZnBr_2_ precipitation is not expected. As a proof of this conclusion, neither the bulk XRD nor the surface-sensitive XPS results (see later) show any indication of the formation of ZnBr_2_ crystal or the presence of Br^−^ ions, which means that the high solubility and the washing procedure resulted in only ZnO/Zn(OH)_2_. There is also the possibility of forming soluble complexes between Zn^2+^ and Br^−^ [12], but our results show no indication of ZnO NP contamination with such complexes. Upon increasing the number of OH^−^ ions, the aggregation behavior orients itself from the spherical shape to the elongated shape. At high pH, ZnO carries negative charges, and hence, the cationic surfactant will preferentially adsorb on the nanoparticles and present constrain on the growth direction and hence on the shape. Such constrain is absent when anionic or non-ionic surfactants are used [1-4]. It is worth mentioning here that beside the phase behavior change (minimum droplet interaction and lower precursor sensitivity), the presence of small amount of non-ionic surfactants (10 mol%) with the majority of cationic surfactants (90 mol%) is expected to lower the polarity of the surfactant films, and hence, a slightly milder interaction with the ions is expected.

**Figure 1:**
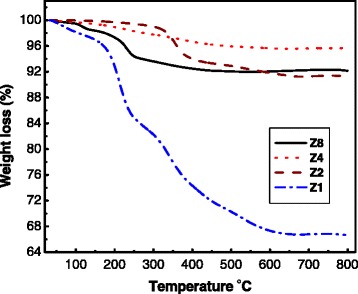
**TGA curves for the as-synthesized ZnO NPs.**

**Figure 2:**
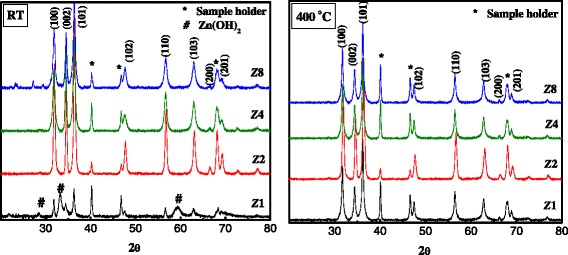
**XRD patterns for room temperature (RT) and 400** **°C** **-calcined ZnO NPs.**

TGA of the as-synthesized ZnO samples is shown in Figure 1. Three main decomposition steps can be observed: the first weight loss step for the four samples is up to about 180°C, which demonstrates the dehydration of surface-adsorbed water. The second step, at 180 °C to 330°C, is related to the decomposition of Zn(OH)_2_ to ZnO. The third weight loss, at 330°C to 540°C, is referred to the decomposition of the remaining adsorbed surfactants [13]. The plateau at *T* > 540°C corresponds to the formation of the ZnO NPs [14]. Based on the weight loss data from the TGA results, the percentages for ZnO/Zn(OH)_2_ are 84/16%, 99/1%, 99/1%, and 96/4% for ***Z***1, ***Z***2, ***Z***4, and ***Z***8, respectively. Obviously, the thermogravemetric study shows that a gradual weight loss decreases with increasing the concentration of NaOH up to ***Z***4 and then increases for ***Z***8. This is referred to the enhancement of the conversion from hydroxide to oxide form upon increasing the NaOH concentration. The different behavior of ***Z***8 could be a result of the organic phase contaminations resulting from the onset of microemulsion breakdown at such high NaOH concentration. For example, the relatively large weight loss for ***Z***1 rather than the other samples is attributed to the presence of larger amount of Zn(OH)_2_ which is confirmed from the XRD results (Figure 2).

The diffraction peaks in Figure 2 which are located at 31.86°, 34.53°, 36.54°, 47.57°, 56.78°, 62.96°, 66.80°, and 69.30° have been indexed as hexagonal wurtzite phase of ZnO [15,16] (JCPDS: 36–1451). The diffraction pattern of the synthesized ZnO NPs (***Z***1) at room temperature (Figure 2) shows diffraction peaks at 2*θ* = 28.0°, 33.2°, and 59.0° which are attributed to the presence of some Zn(OH)_2_ [17]. Those peaks completely disappeared upon calcination at 400°C, which confirms its dissociation to a phase pure ZnO (Figure 2). Using Scherrer's formula, the average crystallite sizes were calculated and reported in Table 1.

**Table 1:** **Specific surface area,** ***S***_**BET**_ **, BJH average pore diameter and volume,** ***D***_**p**_ **and** ***V***_**p**_ **, crystallite size,** ***l*** **, and particle size,** ***D***

**Sample**	***S***_**BET**_ **(m** ^**2**^ **g** ^**−1**^ **)**	***D***_**p**_ **(nm)**	***V***_**p**_ **(cm** ^**3**^ **g** ^**−1**^ **)**	***L*** **(nm)** ^**a**^	***D*** **(nm)**
***Z***1	57	10.7	0.15	49	110
***Z***2	76	23.9	0.46	46	70, 1,080^b^
***Z***4	53	17.3	0.23	31	35, 90^b^
***Z***8	21	10.9	0.06	32	57

^a^Measured at the more intense (101) peak. ^b^Particle diameter and length, respectively.

**Figure 3:**
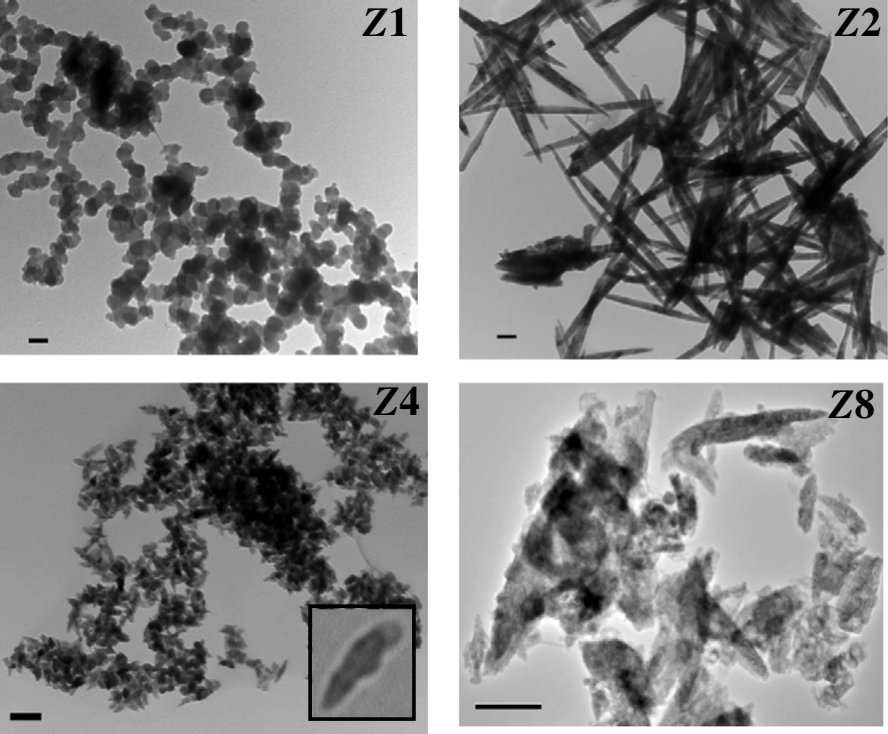
**TEM images of the as-prepared ZnO NPs of different morphologies.** The scale bar is 200 nm.

**Figure 4:**
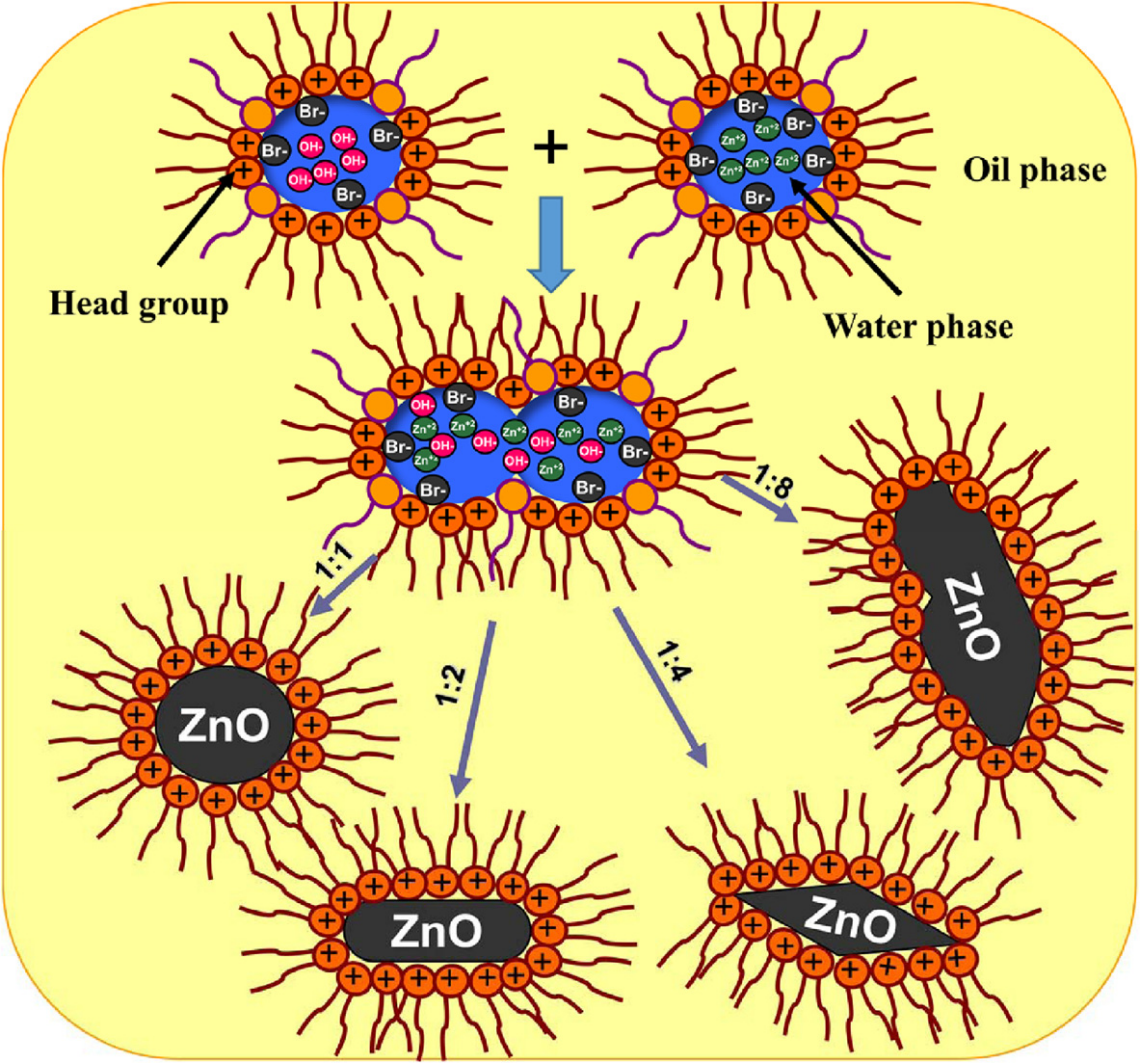
**Zn** ^**+2**^ **, Br** ^**−**^ **, and OH** ^**−**^ **ion interaction and effect of [Zn** ^**2+**^ **]:[OH** ^**−**^ **] ratio on ZnO NP morphologies.**

The morphology of the ZnO NPs was investigated by TEM (Figure 3). At 1:1 Zn precursor:NaOH (***Z***1), the NPs preferred a well-organized non-aggregated spherical shape with an average diameter of 110 nm. At 1:2 Zn precursor:NaOH (***Z***2), the produced NPs preferred a needle-like shape with high aspect ratio (average 70 nm in diameter and 1,080 nm in length). At 1:4 Zn precursor:NaOH (***Z***4), the needle-like shape transformed to an acicular shape with an average diameter of 55 nm. Further increase in the precipitating agent concentration gives irregular shape as shown in sample ***Z***8. This change of shape as a function of [NaOH] is attributed to OH^−^ ion concentration which contributes to the nucleation process [18]. The relatively larger nanoparticles when compared with those prepared in other microemulsion systems [1-4] means that the growth is more dominant than the nucleation process. The interaction of the reactants (Zn^+2^ and OH^−^) with the positive interfacial quaternary ammonium groups and Br^−^ counter ions could result in such larger sizes. This effect is explained by the fact that Zn^2+^ ions are repelled from the surfactant film region while the OH^−^ ions are attracted to the, mainly cationic, surfactant film. On the other hand, the Br^−^ ions will be attracted to the Zn^2+^ ions much more than the OH^−^ ions. This is due to the higher polarizibility and hence the smaller charge density of the large Br^−^ ions [9,10]. In other words, in the competition between the Br^−^ and OH^−^ ions to bind with the Zn^2+^, the Br^−^ ions are certainly a winner. It is worth mentioning that considering the amount of water used for *W* = 18 microemulsion, the cationic surfactant concentration = 0.18 M, and using the valid assumption of the absence of the double-tailed cationic surfactant in the oil, the aqueous concentration of Br^−^ was found to be 5.6 M while the employed aqueous concentration of OH^−^ is in the range of 0.1 to 0.8 M (i.e., the population of the Br^−^ ions in the droplet core is much more than that of OH^−^). This further justifies the winning effect of Zn^+2^-Br^−^ interaction over that of Zn^+2^-OH^−^. Such interaction retard the fast nucleation processes and the slower growth process become the dominating one. The shape, however, is determined by the pH of the solution similar to what can be observed in aqueous solution. It is worth mentioning that monodispersity and porosity of ZnO in aqueous solution are much worse than with those obtained in this study [19]. The cartoon in Figure 4 explains the ion interactions and morphology dependence on Zn^+2^:OH^−^ molar ratio.

**Figure 5:**
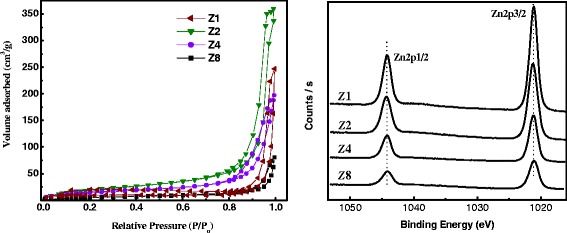
**N2 adorption-desoption isotherms (left) and XPS spectra of Zn 2p (right) for** ***Z*** **1 to** ***Z*** **8 samples.**

All of the N_2_ isotherms can be ascribed as type IV with hysteresis loop of type H3, indicating mesoporous structure with slit-shaped pores (Figure 5). Also, the capillary condensation occurs at high relative pressures, and adsorption-desorption saturation is not significant due to the presence of large mesopores [20]. N_2_ sorpometry measurements revealed that the specific surface area (*S*_BET_), pore diameter (*D*_p_), and the pore volume (*V*_p_) for ZnO NPs are dependent on the concentration of NaOH (Table 1). It is quite obvious that the extent of porosity (*V*_p_: ***Z***2 > ***Z***4 > ***Z***1 > ***Z***8) and not the pore size (*D*_p_: ***Z***2 > ***Z***4 > ***Z***1 ≈ ***Z***8) is the determining factor for the *S*_BET_ (NPs with needle-like shape have the highest *V*_p_). It is also noticeable that the ZnO synthesized in microemulsion possess *V*_p_ much higher than those prepared in other polar solvents such as water or ethanol [19]. The doublet Zn 2p spectrum in Figure 5 shows binding energies of 1,021.1 and 1,044.2 eV, which referred to Zn-2p_3/2_ and Zn-2p_1/2_, respectively, and are indicative of the Zn^2+^ oxidation state in ZnO [21]. The binding energy difference between Zn-2p_3/2_ and Zn-2p_1/2_ is 23.1 eV, which is close to the standard value of ZnO [22]. The variation in the concentration of the precipitating agent did not alter the binding energy values of Zn 2p.

## Conclusions

In summary, well-organized monophasic and monodispersed ZnO NPs with high surface area and porosity (Table 1) and different morphologies (Figure 3) were synthesized using the W/O μ system of relative insensitivity toward precursor addition. This insensitivity is established by replacing 10 mol% of the cationic DDAB surfactant films by the non-ionic Brij®35 surfactants [5,6]. In this short communication, particular focus was given to the role of different microemulsion constituents. Polarizability and electron charge density (which is a function of the ion size) are believed to control the initial interaction between the Zn^+2^ and Br^−^ and/or OH^−^ ions, and hence, they control also the nucleation process. In spite of the retardation to the nucleation, the surface area of the ZnO NPs was found to be high and this is not due to the NP sizes but due to the relatively high porosity.

## References

[1] Sarkar D, Tikku S, Thapar V, Srinivasa RS, Khilar KC (2011). Formation of zinc oxide nanoparticles of different shapes in water-in-oil microemulsion. Colloids Surf, A..

[2] Li X, He G, Xiao G, Liu H, Wang M (2009). Synthesis and morphology control of ZnO nanostructures in microemulsions. J Colloid Interface Sci..

[3] Inoguchi M, Suzuki K, Kageyama K, Takagi H, Sakabe Y (2008). Monodispersed and well-crystallized zinc oxide nanoparticles fabricated by microemulsion method. J Am Ceram Soc..

[4] Yıldırım ÖA, Durucan C (2010). Synthesis of zinc oxide nanoparticles elaborated by microemulsion method. J Alloy Comp..

[5] Bumajdad A, Eastoe J, Zaki MI, Heenan RK, Pasupulety L (2007). Generation of metal oxide nanoparticles in optimised microemulsions. J Coloid Interface Sci..

[6] Bumajdad A, Eastoe J, Nave S, Steytler DC, Heenan RK, Grillo I (2003). Compositions of mixed surfactant layers in microemulsions determined by small-angle neutron scattering. Langmuir..

[7] Krishnan D, Pradeep T (2009). Precursor-controlled synthesis of hierarchical ZnO nanostructures, using oligoaniline-coated Au nanoparticle seeds. J Cryst Growth..

[8] Ramdas BK, Bhaskar DK (2008). Nanoreactors for nanostructured materials. Int J Chem React Eng..

[9] Thompson RA, Allenmark S (1992). Factors influencing the micellar catalyzed hydrolysis of long-chain alkyl betainates. J Colloid Interface Sci..

[10] Oh S-G, Kizling J, Holmberg K (1995). Microemulsions as reaction media for synthesis of sodium decyl sulfonate 2. Role of ionic surfactants. Colloids Surf, A.

[11] Patnaik P (2002). Handbook inorganic chemicals.

[12] Yang MM, Crerar A, Irish DE (1988). Raman spectral studies of aqueous zinc bromide solutions to 300°C at pressures of 9 MPa. J Solution Chem..

[13] Raoufi D (2013). Synthesis and microstructural properties of ZnO nanoparticles prepared by precipitation method. Renew Energy..

[14] Chen C, Liu P, Lu C (2008). Synthesis and characterization of nano-sized ZnO powders by direct precipitation method. Chem Eng J..

[15] Zhou J, Zhao F, Wang Y, Zhang Y, Yang L (2007). Size-controlled synthesis of ZnO nanoparticles and their photoluminescence properties. J Lumin..

[16] Chen Y, Yu R, Shi Q, Qin J, Zheng F (2007). Hydrothermal synthesis of hexagonal ZnO clusters. Mater Lett..

[17] Chakraborty S, Kumbhakar P (2013). Observation of exciton–phonon coupling and enhanced photoluminescence emission in ZnO nanotwins synthesized by a simple wet chemical approach. Mater Lett..

[18] Peng ZA, Peng X (2001). Mechanisms of the shape evolution of CdSe nanocrystals. J Am Chem Soc..

[19] Bagabas A, Alshammari A, Aboud M, Kosslick H (2013). Room-temperature synthesis of zinc oxide nanoparticles in different media and their application in cyanide photodegradation. Nanoscale Res Lett..

[20] Singh S, Barick KC, Bahadur D (2013). Shape-controlled hierarchical ZnO architectures: photocatalytic and antibacterial activities. CrystEngComm..

[21] Wahab R, Ansari SG, Kim YS, Seo HK, Kim GS, Khang G (2007). Low temperature solution synthesis and characterization of ZnO nano-flowers. Mater Res Bull..

[22] Zhang G, Morikawa H, Chen Y, Miura M (2013). In-situ synthesis of ZnO nanoparticles on bamboo pulp fabric. Mater Lett..

